# Supplementary material for: Regulation of Cellular Senescence Is Independent from Profibrotic Fibroblast-Deposited ECM
Source: Cells. 2021 Jun 29;10(7):1628. doi: 10.3390/cells10071628 (PMC8307656; doi:10.3390/cells10071628)
Supplement: Supplementary file 1 [file cells-10-01628-s001.zip › For XML Supplementary.pdf]

cells

MDPI

Supplementary Data

Regulation of Cellular Senescence Is Independent from Profibrotic Fibroblast-Deposited ECM

Kaj E.C. Blokland [1,2,3,4], Habibie Habibie [2,5,6], Theo Borghuis [1,2], Greta J. Teitsma [1,2], Michael Schuliga [3], Barbro N. Melgert [1,2,5], Darryl A. Knight [3,4,7], Corry-Anke Brandsma [1,2], Simon D. Pouwels [1,2,8] and Janette K. Burgess [1,2,*]

Senescence-associated β-galactosidase protocol

Cellular senescence was assessed using a previously described protocol for senescence-associated β-galactosidase (SA-β-Gal) staining [43]. Fixed fibroblasts were washed with PBS and SA-β-Gal staining solution was added. Plates were incubated in a dry incubator at 37 °C for 16 h. Staining solution was aspirated before washed with PBS. PBS containing 1 μg/mL 4′,6-diamidino-2-phenylindole (DAPI; Sigma-Aldrich) was added and plates were incubated in the dark for 10 min, washed and stored in 70% glycerol in PBS at 4 °C. Plates were imaged using a TissueFAXS automated analysis system (TissueGnostics, Vienna, Austria). Brightfield (SA-β-Gal) and DAPI images were exported using TissueFAXS Viewer 7.0 (TissueGnostics) and analysed in FIJI [44,45]. To count the number of nuclei the DAPI images were opened in FIJI. First a background subtraction was performed with a rolling ball radius of 50.0 pixels followed by a threshold setting from 35 to 255. After the image was converted to a mask a "watershed segmentation" was run. Finally, "Analyse particles" was run with size setting of "8-infinity". To count the number of SA-β-Gal positive cells the brightfield images were opened in FIJI. First the images were colour deconvoluted with vector settings "0.960,0.230,0.150,0.150,0.850,0.500,0.000,0.000,0.000" to extract the SA-β-Gal positive staining. Analyse Particles was then run on the image with the SA-β-Gal staining with size settings: "125 to infinity" and a circularity of "0.20 - 1.00". Total cell numbers and SA-β-Gal positive cells were used to calculate the percentage of SA-β-Gal positive cells. To improve the quality of the images, all SA-β-Gal images in S1 and S7 have undergone the following modifications. First, we performed a white balance correction using the ImageJ macro "White balance correction 1.0" (http://pmascalchi.github.io/ImageJ_Auto-white-balance-correction/), then we enhanced the brightness (-20%), contrast (+20%) and sharpness (+50%) using the build-in function of Microsoft PowerPoint.

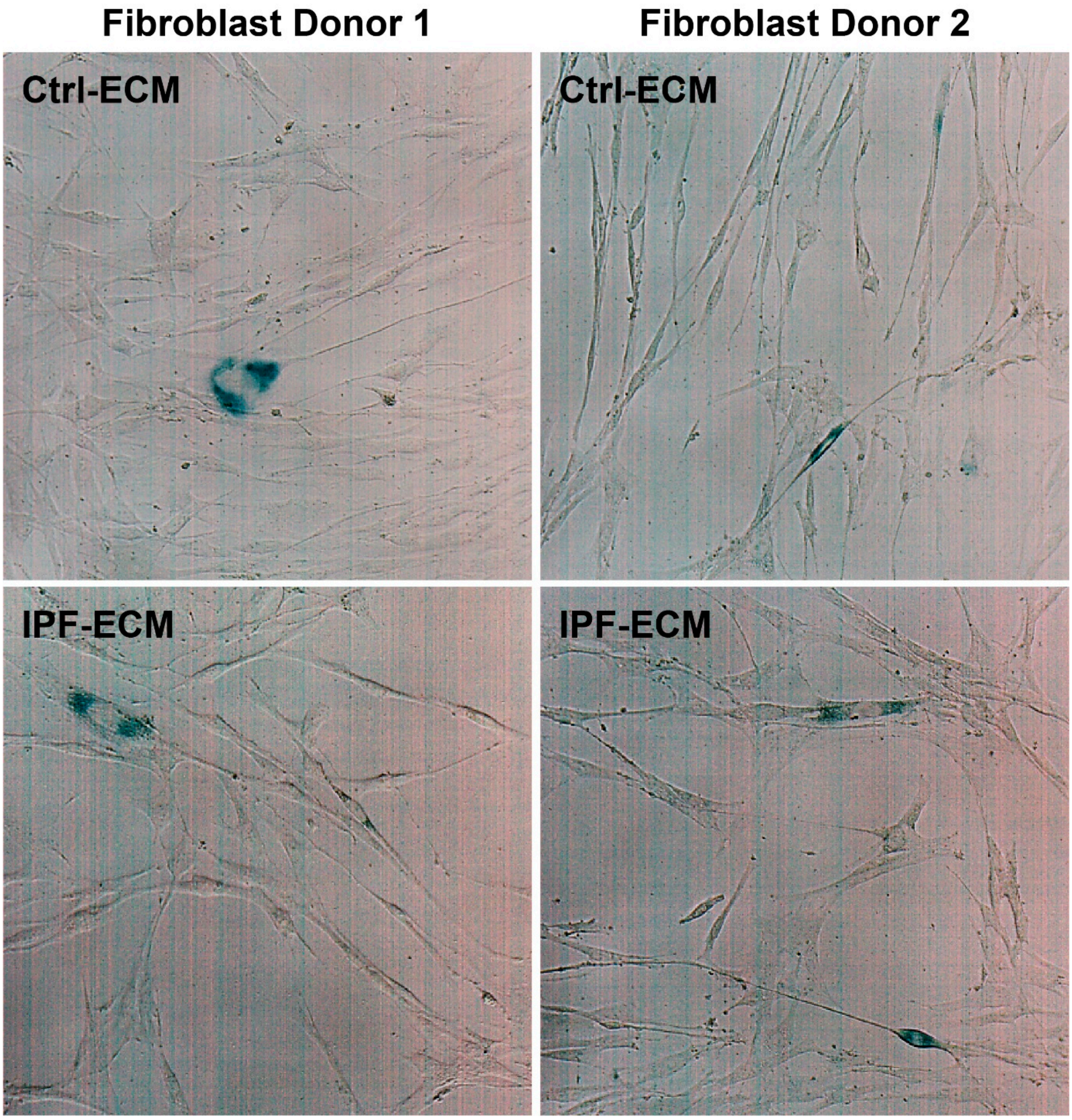

Figure S1. SA-β-Gal positive cells cultured on Ctrl- and IPF derived ECM. Ctrl-LFs were cultured for up to three days on Ctrl or IPF derived ECM and SA-β-Gal positive (blue) cells were visualised using a TissueFAXS and counted using ImageJ. Illustrated photographs are representative images of in total 6 unique donors.

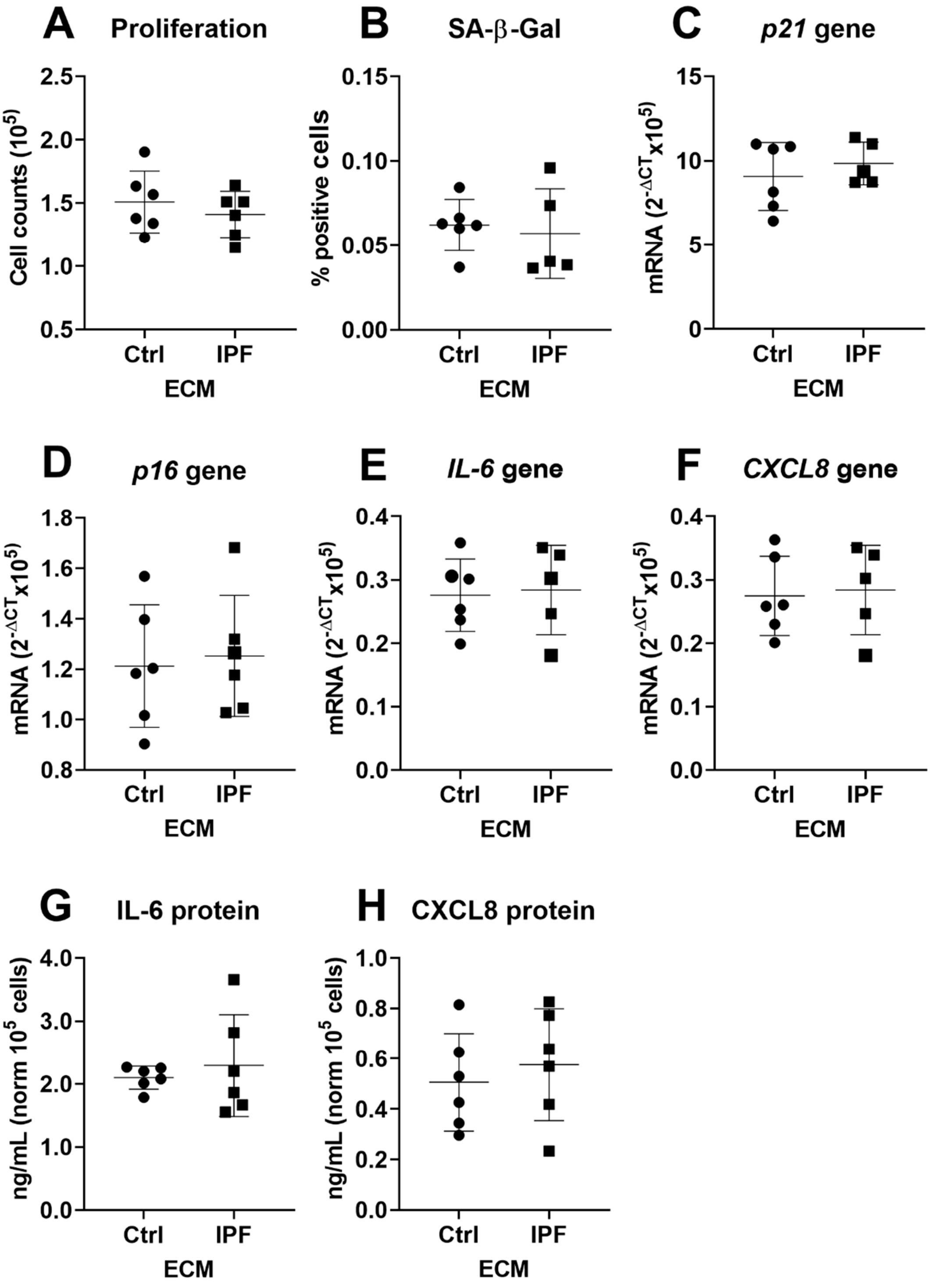

Figure S2. Markers of senescence in Ctrl-LF cultured on Ctrl- or IPF-derived ECM. Ctrl-LFs were cultured for up to three days on Ctrl or IPF derived ECM and proliferation was assessed by cell enumeration (**A**), and SA-β-Gal positive cells were counted (**B**). Panel (**C**) and (**D**) demonstrate cell-cycle inhibitors $p21^{Waf1/Cip1}$ and $p16^{Ink4a}$ after three days of culture. Panel (**E**–**H**) gene expression and protein secretion of known SASP factors IL-6 and CXCL8. Gene expression data were normalised against 18S and were expressed as $2^{-\Delta CT}$ x 10^5 (n = 5–6). Levels of cytokine production in supernatant were normalised to total cell number expressed as ng/mL per 10^5 cells (n = 5–6). Wilcoxon matched-pairs signed rank test was used to measure difference between Ctrl and IPF and was considered significant at $p < 0.05$.

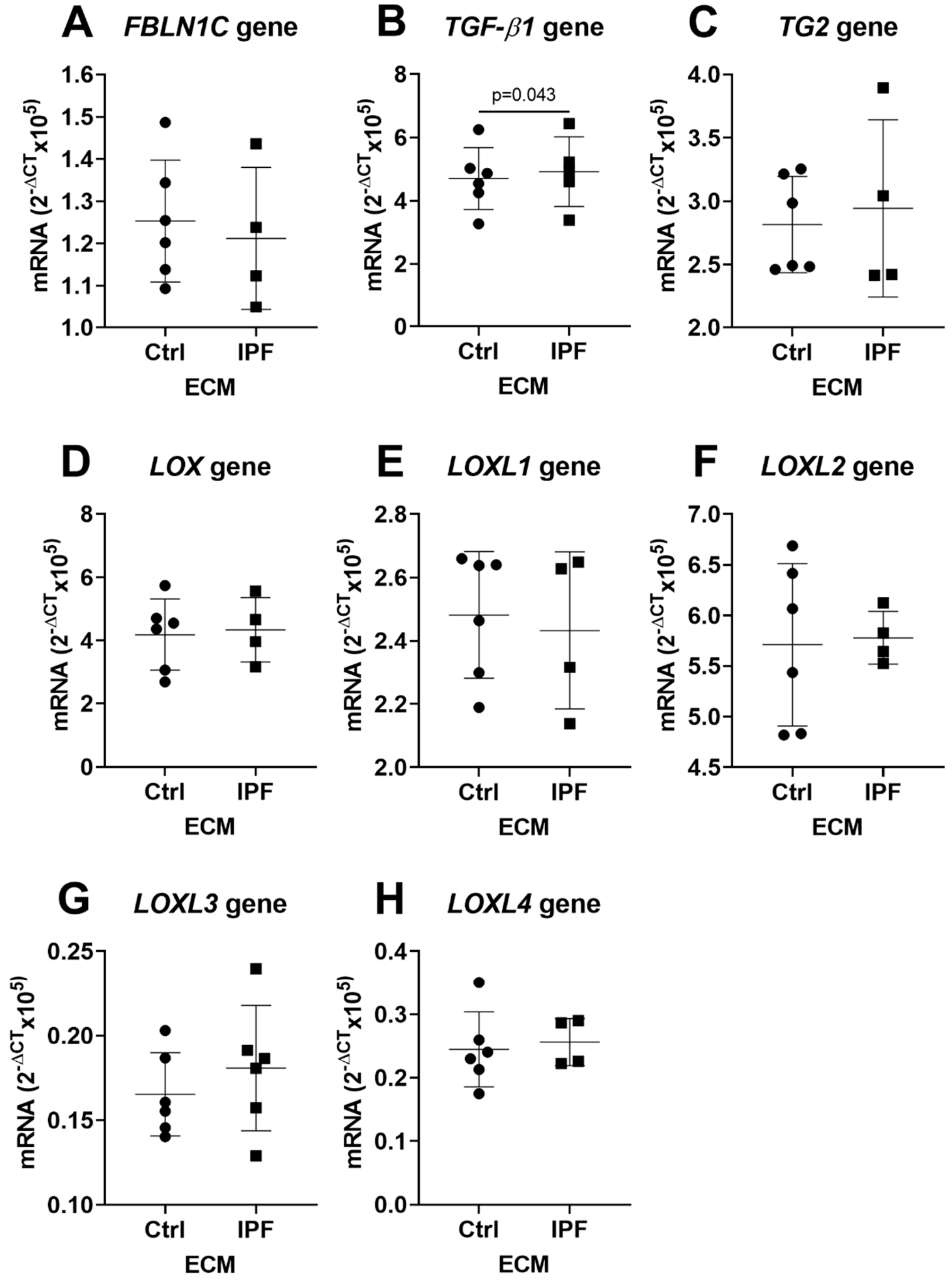

Figure S3. Fibrosis-associated and crosslink gene expression. Ctrl-LFs were cultured for up to three days on Ctrl or IPF derived ECM before expression levels of Fbln1c, TGF-β1, TG2, LOX and LOXL1–4 were assessed (**A**–**H**). Gene expression data were normalised against 18S and were expressed as $2^{-\Delta CT}$ x 10^5 (n = 5–6). Wilcoxon matched-pairs signed rank

test was used to measure difference between Ctrl and IPF and was considered significant at $p < 0.05$.

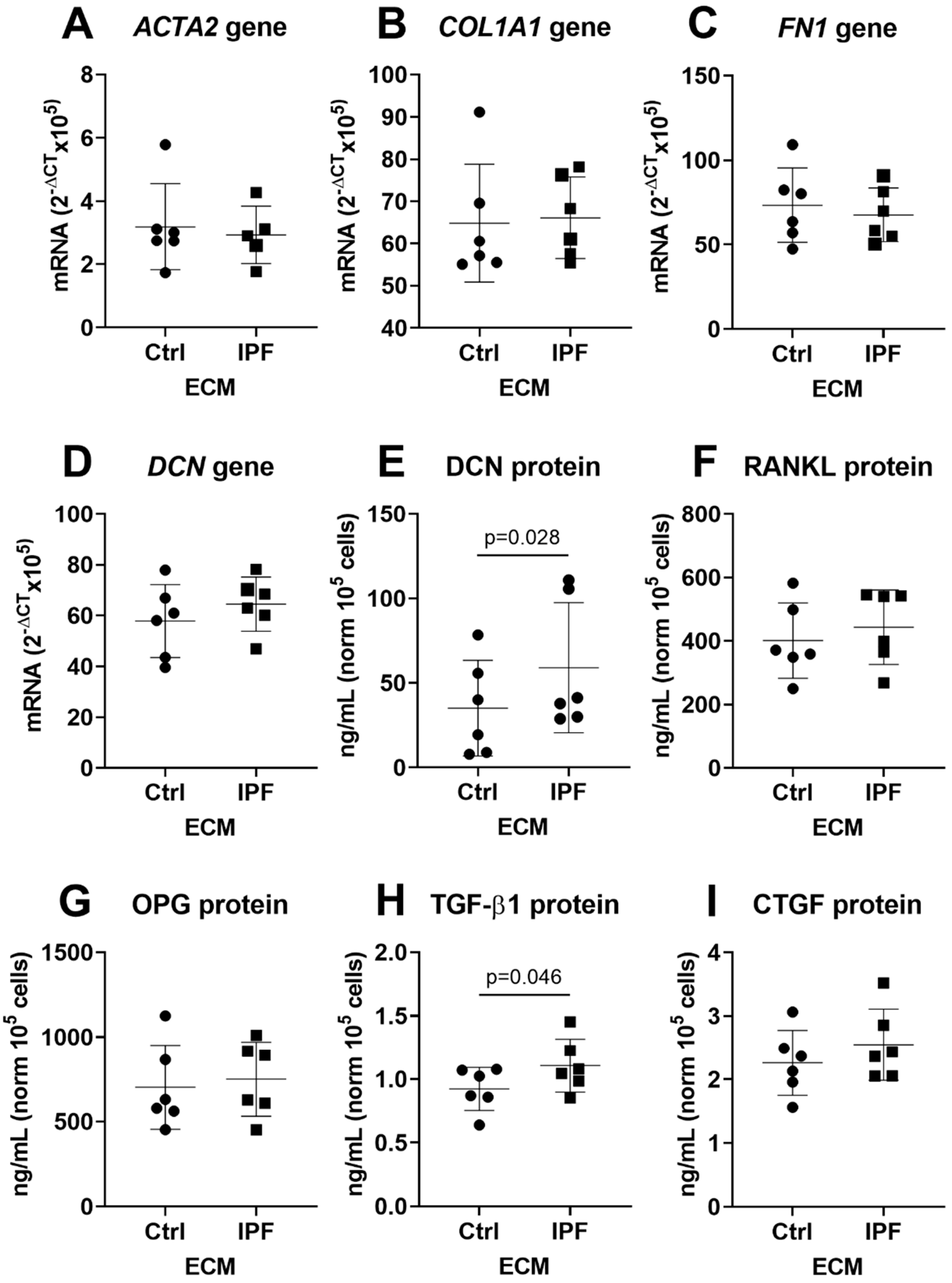

Figure S4. Fibrosis-associated gene expression and secretion of pro-fibrotic cytokines. Ctrl-LFs were cultured for up to three days on Ctrl or IPF derived ECM before expression levels of ACTA2, COL1α1, FN1 and DCN were assessed (**A**–**D**). Panel (**F**–**I**) shows protein secretion of known fibrotic factors DCN, RANKL, OPG, TGF-β1 and CTGF. Gene expression data were normalised against 18S and were expressed as $2^{-\Delta CT} \times 10^5$ (n = 5–6). Levels of cytokine production in supernatant were normalised to total cell number expressed as ng/mL per 10^5 cells (n = 5–6). Wilcoxon matched-pairs signed rank test was used to measure difference between Ctrl and IPF and was considered significant at $p < 0.05$.

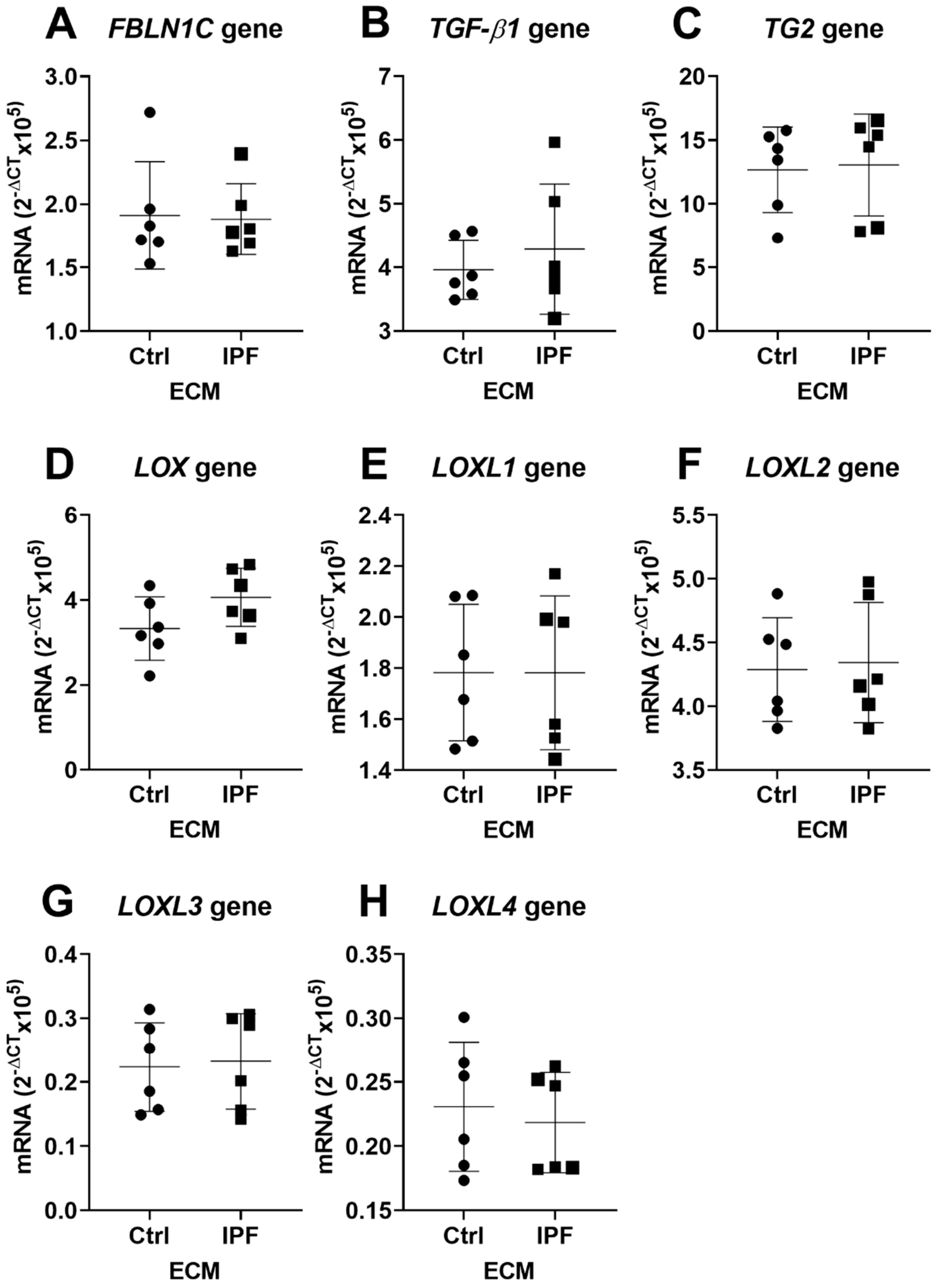

Figure S5. Fibrosis-associated and crosslink gene expression. Ctrl-LFs were cultured for up to three days on Ctrl or IPF derived ECM before expression levels of Fbln1c, TGF-β1, TG2, LOX and LOXL1–4 were assessed (**A**–**H**). Gene expression data were normalised against 18S and were expressed as $2^{-\Delta CT}$ x 10^5 (n = 5–6). Levels of cytokine production in supernatant were normalised to total cell number expressed as ng/mL per 10^5 cells (n = 5–6). Wilcoxon matched-pairs signed rank test was used to measure difference between Ctrl and IPF and was considered significant at $p < 0.05$.

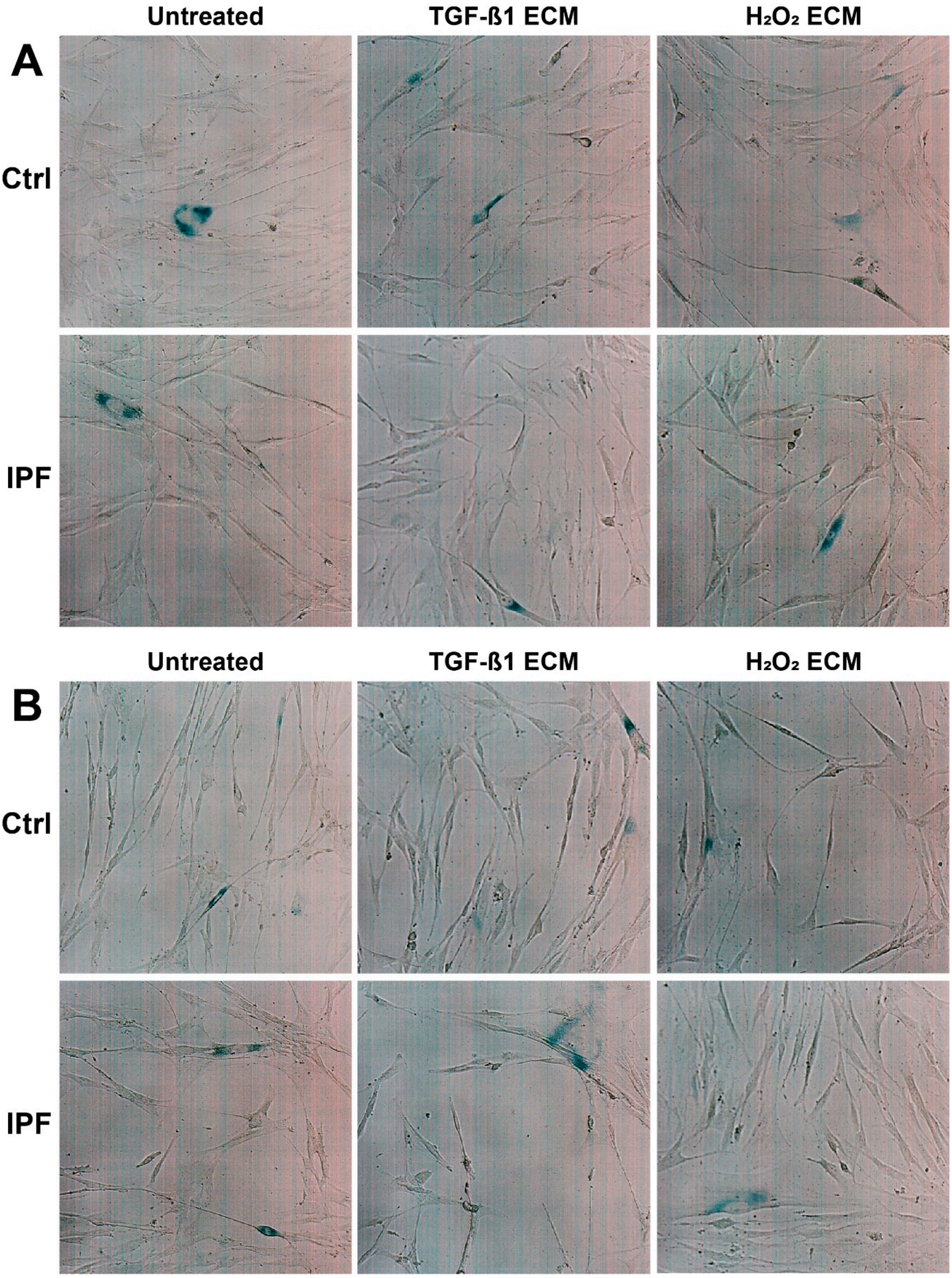

Figure S6. SA-β-Gal positive cells cultured on Ctrl- and IPF derived ECM that received treatment. Ctrl-LFs were cultured for up to three days on Ctrl or IPF derived ECM and SA-β-Gal positive (blue) cells were visualised using a TissueFAXS. Illustrated photographs are representative images of in total 6 unique donors. Control and IPF untreated are the same images as in Figure S1.

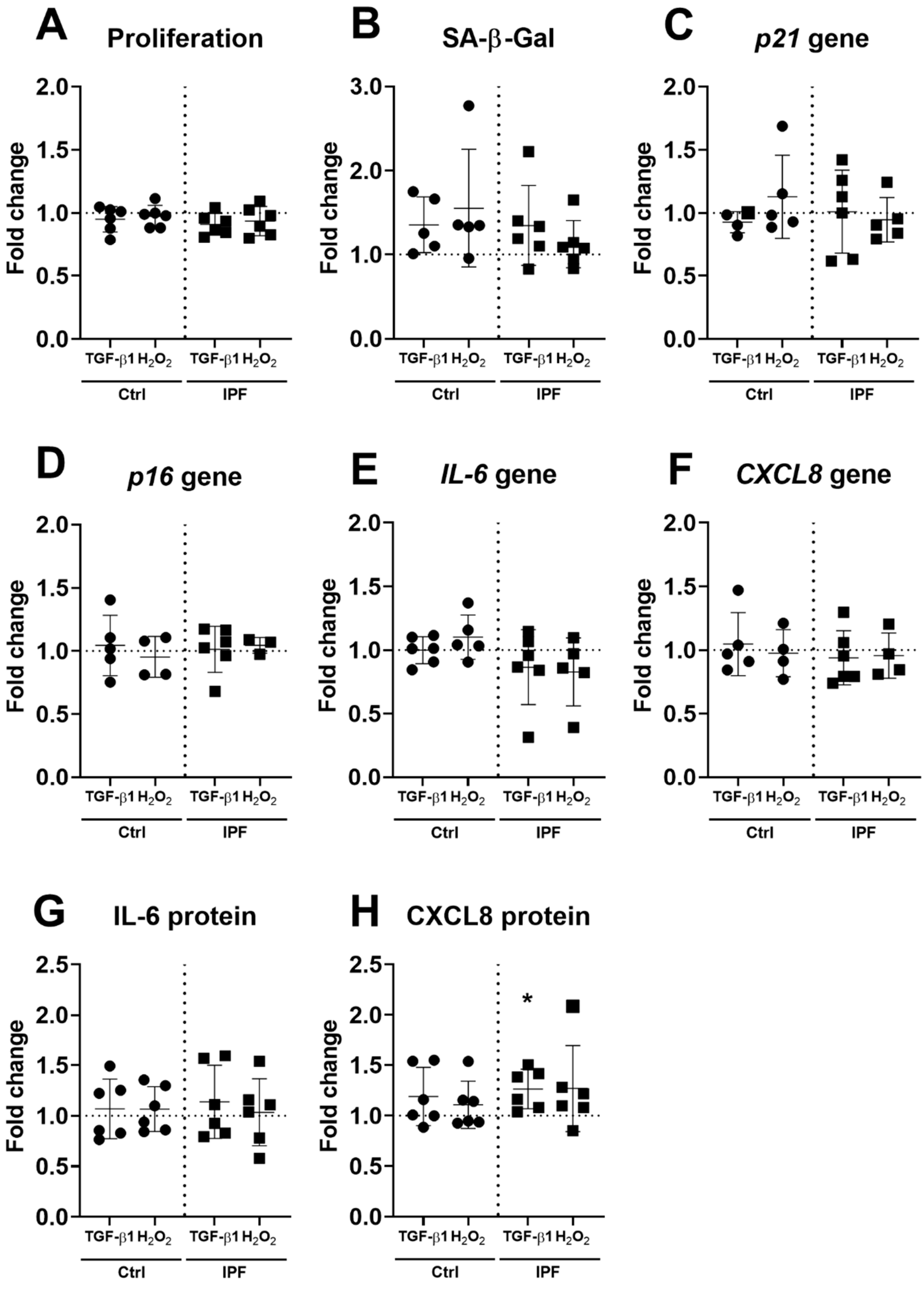

Figure S7. Markers of senescence in Ctrl-LFs cultured on treatment-derived ECM. Ctrl-LFs were cultured for up to three days on Ctrl or IPF derived ECM that received treatment with TGF-β1 or H_2O_2 and proliferation was assessed by cell enumeration (**A**), and SA-β-Gal positive cells were counted (**B**). Panel (**C**) and (**D**) demonstrates cell-cycle inhibitors $p21^{Waf1/Cip1}$ and $p16^{Ink4a}$ after three days of culture. Panel (**E–H)** gene expression and protein secretion of known SASP factors IL-6 and CXCL8. Both gene expression and levels of cytokine production were normalised as described before and expressed as fold change to their respecting Ctrl- or IPF-derived ECM without treatment (n = 5–6). Data were analysed using repeated measures one-way ANOVA or if data points were missing a mixed-effects analysis (REML) and considered significant at * $p < 0.05$.

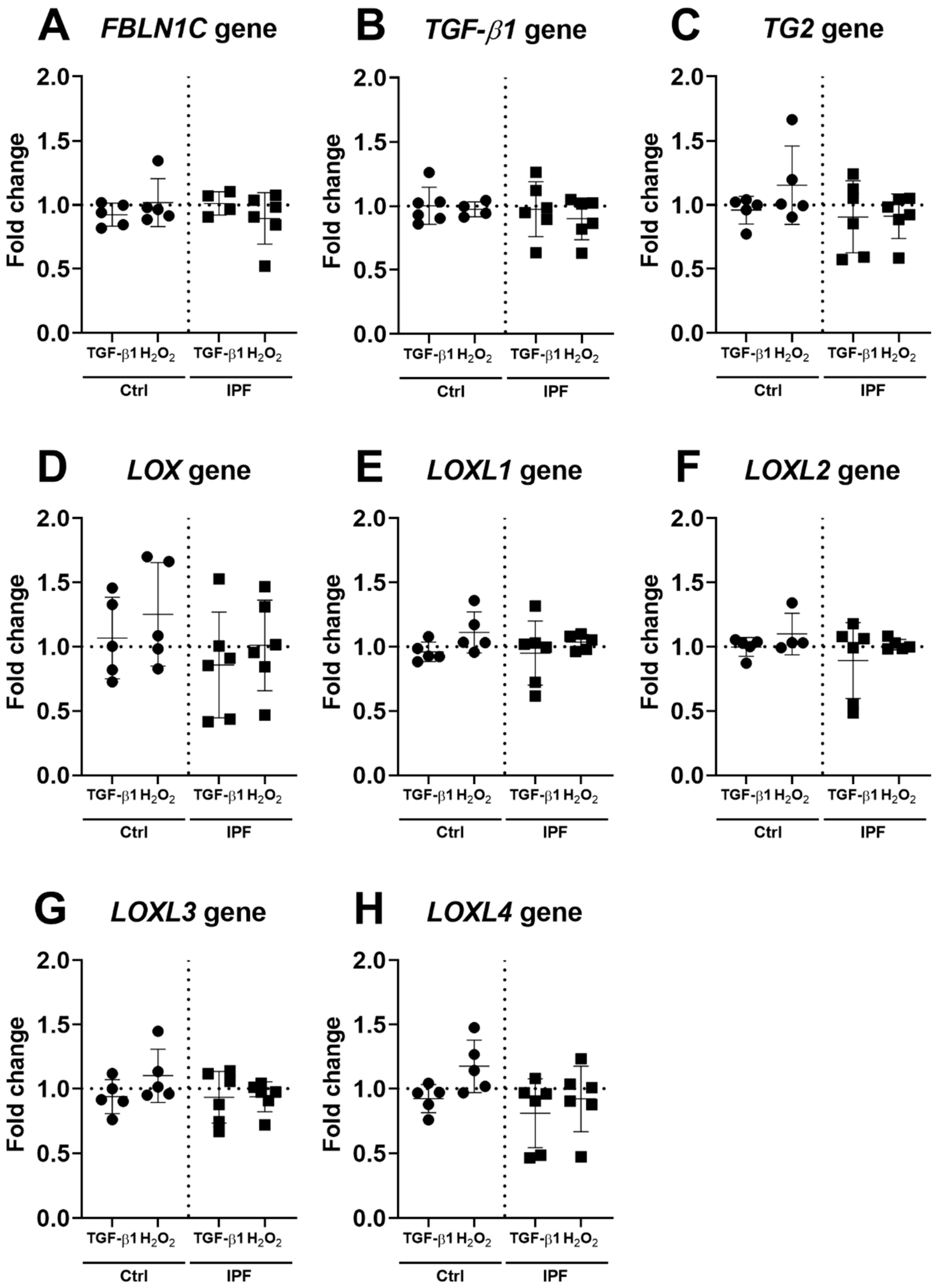

Figure S8. Fibrosis-associated and crosslink gene expression. Ctrl-LFs were cultured for up to three days on Ctrl or IPF derived ECM before expression levels of Fbln1c, TGF-β1, TG2, LOX and LOXL1–4 were assessed (**A**–**H**). Data were normalised as described before and expressed as fold change to their respecting Ctrl- or IPF-derived ECM without treatment (n = 5–6). Data were analysed using repeated measures one-way ANOVA or if data points were missing a mixed-effects analysis (REML) and considered significant at * $p < 0.05$.

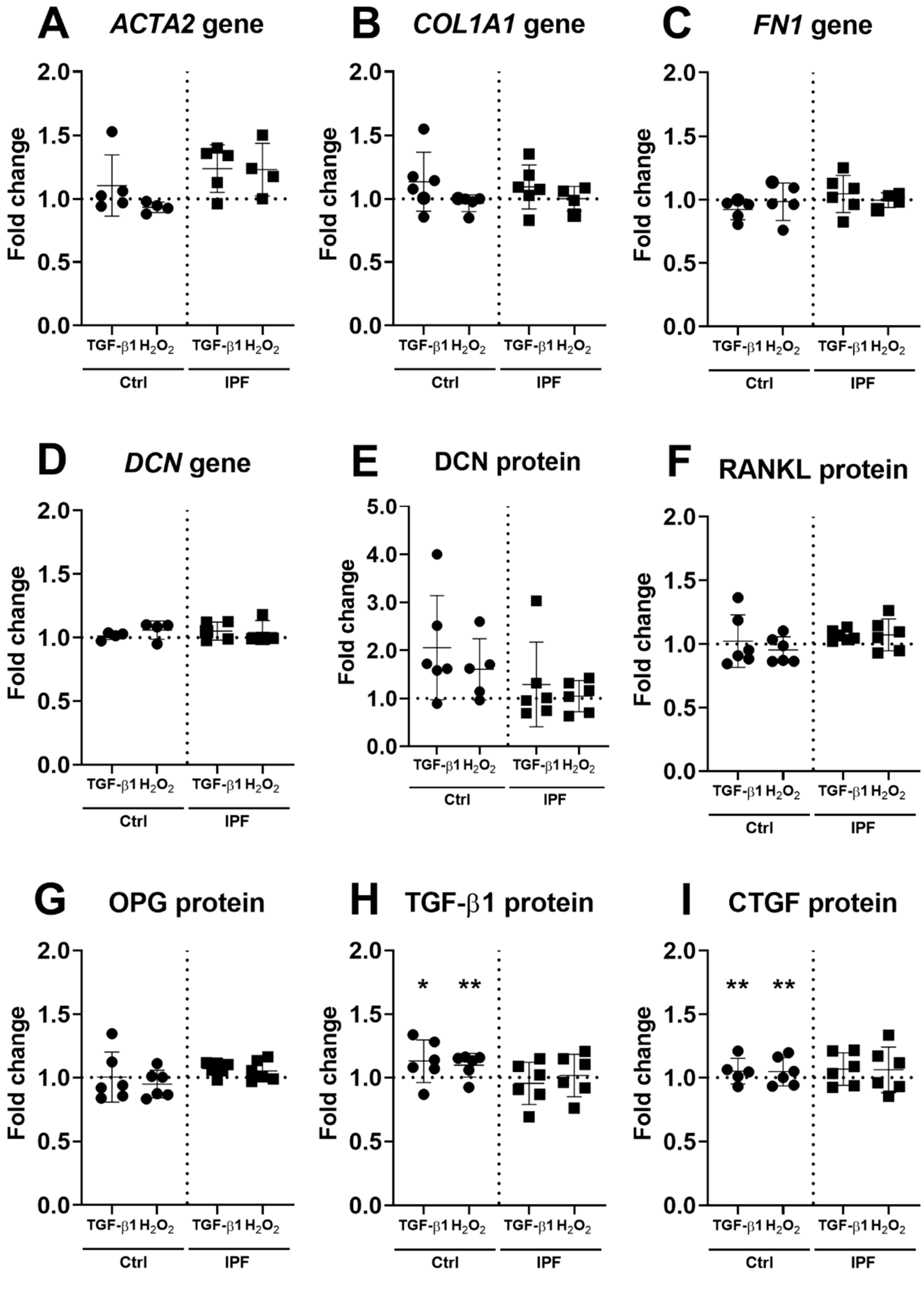

Figure S9. Fibrosis-associated gene expression and secretion of pro-fibrotic cytokines. Ctrl-LFs were cultured for up to three days on treatment-derived Ctrl- or IPF-ECM before expression levels of ACTA2, COL1α1, FN1 and DCN were assessed (**A**–**D**). Panel F-I shows protein secretion of known fibrotic factors DCN, RANKL, OPG, TGF-β1 and CTGF. Both gene expression and levels of cytokine production were normalised as described before and expressed as fold change to their respecting Ctrl- or IPF ECM without treatment (n = 4–6). Data were analysed using repeated measures one-way ANOVA or if data points were missing a mixed-effects analysis (REML) and considered significant at * $p < 0.05$.

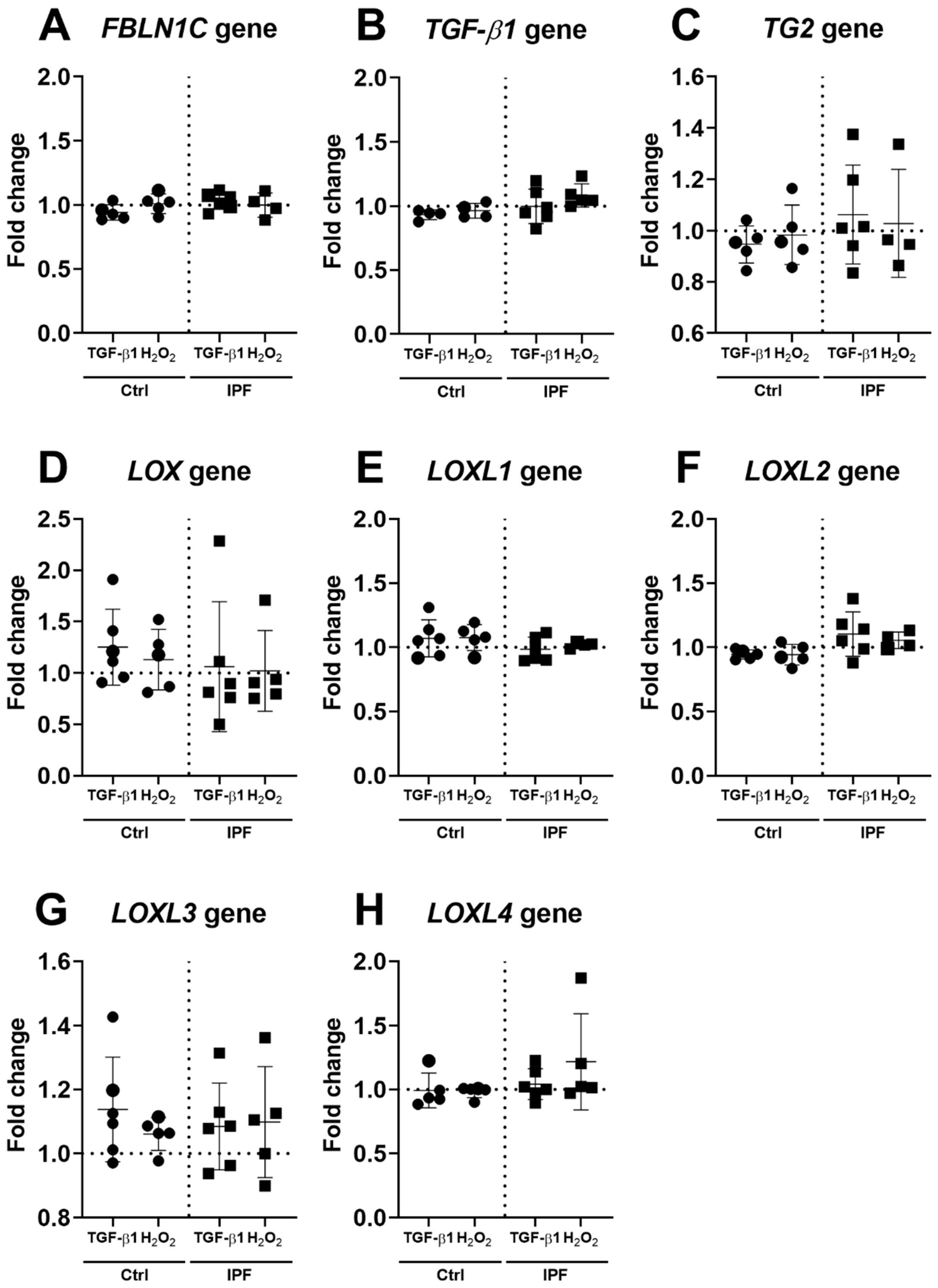

Figure S10. Fibrosis-associated and crosslink gene expression. Ctrl-LFs were cultured for up to three days on Ctrl or IPF derived ECM before expression levels of Fbln1c, TGF-β1, TG2, LOX and LOXL1–4 were assessed (**A**–**H**). Data were normalised as described before and expressed as fold change to their respecting Ctrl- or IPF-derived ECM without treatment (n = 4–6). Data were analysed using repeated measures one-way ANOVA or if data points were missing a mixed-effects analysis (REML) and considered significant at * $p < 0.05$.
